# Lightweight Biometric Sensing for Walker Classification Using Narrowband RF Links

**DOI:** 10.3390/s17122815

**Published:** 2017-12-05

**Authors:** Tong Liu, Zhuo-qian Liang

**Affiliations:** 1 Department of Electronics Engineering, Huizhou University, Huizhou 516001, China; 2 College of Information Science and Technology, Jinan University, Guangzhou 510632, China; tliangzq@jnu.edu.cn

**Keywords:** biometric sensing, walker classification, ubiquitous RF links

## Abstract

This article proposes a lightweight biometric sensing system using ubiquitous narrowband radio frequency (RF) links for path-dependent walker classification. The fluctuated received signal strength (RSS) sequence generated by human motion is used for feature representation. To capture the most discriminative characteristics of individuals, a three-layer RF sensing network is organized for building multiple sampling links at the most common heights of upper limbs, thighs, and lower legs. The optimal parameters of sensing configuration, such as the height of link location and number of fused links, are investigated to improve sensory data distinctions among subjects, and the experimental results suggest that the synergistic sensing by using multiple links can contribute a better performance. This is the new consideration of using RF links in building a biometric sensing system. In addition, two types of classification methods involving vector quantization (VQ) and hidden Markov models (HMMs) are developed and compared for closed-set walker recognition and verification. Experimental studies in indoor line-of-sight (LOS) and non-line-of-sight (NLOS) scenarios are conducted to validate the proposed method.

## 1. Introduction

Acquiring biometric information represented by the physiological and behavioral attributes of human beings has important significance in many security systems and authentication applications [1]. Measurable, stable, and distinctive biometrics exhibit robust connections to individuals, and such traits are advantageous in data–object association based multiple targets tracking and behavior analysis [2,3]. Conventional biometrics sensing and classification techniques have not only made many new advances and widespread applications, but have also developed computational intelligence in emotional analysis [4], health diagnosis [5,6], and soft biometrics [7,8].

From the perspective of the degree of cooperation of the subjects in the phase of feature acquisition, the existing systems can be categorized into constrained and unconstrained manners [9,10]. In the constrained sensing-based systems, the capture of physiological traits such as fingerprint [11], palm print [12], and iris [13] depends on high-quality and short-range sensors. These types of biometrics have been proven to be unique and consistent for individual matching, and some have been applied in the fields of border control and smart ID cards. In addition, with the continuing advances in sensors and pattern recognition technologies, a series of behavioral biometric sensing using wearable devices have emerged, such as electroencephalography (EEG) [14], finger-vein [15], and gait [16] feature acquisitions with wearable sensors. However, the above-described approaches require physical contacts and are subject to highly-controlled environments. Biometric sensing in the context of a free environment and without deliberate cooperation will help to enhance the system’s usability and practical range of applications [10]. In the field of large-scale monitoring, the unconstrained manner is more likely to be the only feasible solution for access control and object tracking.

In unconstrained biometric capture, the physiological and behavioral traits are collected far away from the sensor. Various visible and thermal camera-based vision techniques have been applied to the acquisition of face and gait features [9]. The rapid developments of face identification systems are capable of recognizing and verifying personal identity in a passive and non-intrusive manner [17]. Human gait is comprised of temporal patterns of walking, and has been proven to be unique for each individual [18]. Gait recognition has received a great deal of attention due to its capability of being performed in long-distance surveillance applications and without body-invasive device or user cooperation. However, several problems still plague the two-dimensional (2D) and three-dimensional (3D) modeling of facial and gait features [19,20], including insufficient resolution, robust feature detection, angle correction, landmark registration, and partial occlusions.

Recent trends in wireless communication technology have shown great promise for providing ubiquitous wireless propagation in personal and public areas. The point-to-point (P2P) narrowband radio frequency (RF) links are conventionally employed for networking and information transportation. Exploring and exploiting the functional reuse of existing RF links from data transmission to activity measurement tools is the continuation of big data and Internet of Things (IoT) [21]. The newly proposed device-free radio vision uses the shadow fading characteristics of RF signals transmitted between the network nodes to infer the presence and motion state of internal targets in the environment [22]. Through the distributed deployment, the multiplexing sensing mode can be formed, which is helpful to the reference structure tomography for attenuation imaging induced by the presence of moving objects. In particular, compared with the traditional homogeneous optical imaging and infrared sensing, the RF signal-based sampling and measurement not only have the advantages of wide coverage, feasibility of penetrating obstacles, low cost, low power consumption, and flexible configuration, but also the superiorities of motion-specific sensing and privacy invasiveness. As a result, device-free radio sensing has recently become one of the most promising tomography imaging methods and has formed many practical applications, ranging from target localization and tracking in indoor and outdoor environment [23,24], through to respiratory monitoring [25], motion recognition [26,27], and elderly fall detection [28,29]. However, to the best of our knowledge, little literature has been focused on the biometric sensing of walking modality by using RF links.

In our previous study [30], a vertically deployed RF sensing network was designed to build seven links with different locations of height. Experimental results reveal that the sequential received signal strength (RSS) sampled from a single link will contribute a weak distinctive pattern for walker recognition. The fused biometric feature by all links is capable of offering many rich yet discriminative cues for every person and achieving acceptable performance. However, it is not realistic to deploy a sensing network perpendicular to the ground for practical and large-scale applications.

In this article, we explore the novel use of multiple narrowband RF links to sample biometric traits generated by walking on a constrained path. A three-layer RF sensing network is organized for collecting the perturbations of RSS driven by walking movements of the upper limbs, thighs, and lower legs. The proposed sensing method supports multi-angle, fine-grained, and limb-specific feature capture, and the temporal RSS values can be directly used as the biometric trait generated by an individual. In order to verify the effectiveness of the proposed method, a database with fifteen subjects is established, and the identity of each walker is modeled by vector quantization (VQ) and hidden Markov model (HMM). The optimal parameters for walker classification (i.e., the height of link location, the number of fused links, and the modeling method used) are investigated to improve performance. Experimental studies in indoor line-of-sight (LOS) and non-line-of-sight (NLOS) scenarios are conducted to validate the proposed method.

The rest of this article is organized as follows. Section 2 gives a brief review of related work. Section 3 describes the configuration and implementation of the RF sensing network for biometric acquisition. Section 4 introduces the VQ and HMM based modeling methods for walker recognition and verification. Section 5 presents experimental results and discusses the optimal parameters of sensing configuration. Section 6 concludes the article.

## 2. Related Study

The penetrating capability of electromagnetic signal makes it an important target detection and imaging method for covered environments [31]. There has been widespread adoption of the sophisticated radar techniques such as ultra-wide band (UWB) [32], ultra narrow band (UNB) [33] and multiple input multiple output (MIMO) radar array [34] to infer the presence states of objects by measuring and analyzing the echo signals. Such systems are capable of acquiring coarse-grained silhouettes of the human body and recognizing the identity based on the shape of the body image in a small population group [35]. However, several issues still plague the accurate human body imaging in large-scale scenarios, such as the scatting losses, phase-synchronicity , hybrid multipath effects, and high-cost hardware.

Unlike the radar techniques, RSS-based measurement is less sensitive to scattering loss and the phase of the detected signal, and is more suitable for large-scale, distributed, and networked implementations [36]. The shadowing attenuation separated from the RF link is directly associated with the obstruction of the human body, thus multi-granularity limb-specific sensing can be achieved [37]. Wireless sensor networks (WSNs) are the most commonly used architecture for RF measurements, as a result of the functional expansion of RF links from data transmission to sensing tools. Therefore, the RSS-based biometric sensing will be a complementary method to conventional biometrics systems. In addition, the greatest advantage of RSS measurements is that they can explore and utilize the existing technologies and hardware. Although there have been many works exploring the multiplexing sensing mode of RF links for device-free localization [23,24] and activity recognition [25,27,28,29], the systematic design for acquiring biometrics generated by walking has not yet been formed.

On the basis of our previous study [30], the following extensions have been carried out. First, a sensing method based on a three-layer RF network is proposed, and the combinations of multiple correlated links with different geometric structures are assessed for the discriminant representation of individuals. Secondly, the number of participants in the database is increased to 15 and the HMM is introduced to model the temporal features for each pedestrian. Thirdly, the effectiveness of the proposed sensing approach for close-set walker verification is discussed.

## 3. Sensing Method

Daily walking is the most basic movement of human beings. Although everyone follows a common bipedal walking pattern, early medical research has shown that the way of human walking involves the personal nervous system, the specific composition of bones and muscles, and habit [38,39]. Therefore, human gait is considered as a highly unique feature for biometric representation and is used as a valid criterion for identification.

The most important aspect of a biometric sensing system is how to obtain the most remarkable and discriminative walking feature. According to the research on vision-based gait recognition, structured representation provides a powerful guideline for the design of RF link measurement-based biometric sensing. Johansson’s psychological research shows the identity of an acquaintance and the gender of a person can be distinguished by observing the trajectory of moving light displays (MLDs) attached to the main joints [40,41]. Lee and Grimson divided the extracted human silhouette into symmetrical sub-areas (i.e., head, shoulders, and lower limbs), and fitted them with ellipses [42]. The motion parameters of elliptical joints were used for gait recognition. Zhang et al. proposed a five-link biped locomotion human model for gait representation [43], and showed that the overall movements of the upper limbs, thighs, and calves constitute the important features for gait recognition. Rida et al. presented a human body part selection method by using group Lasso of motion, and the most discriminative features with minimum intra-class variation were studied [44]. Their results demonstrated that multi-limb sensing of walking movements is able to ensure the efficiency of biometric feature collection. In addition, Foster et al. found that gait was more discriminative with horizontal motion than with vertical motion [45]. Thus, lateral sensing of human walking will enable more characterized features.

**Figure 1:**
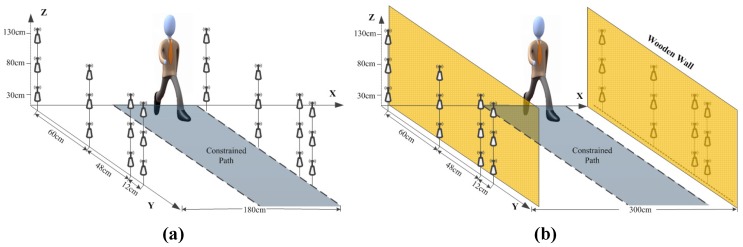
Multi-layer sensing model. (**a**) line-of-sight (LOS) scenario; (**b**) non-line-of-sight (NLOS) scenario.

**Figure 2:**
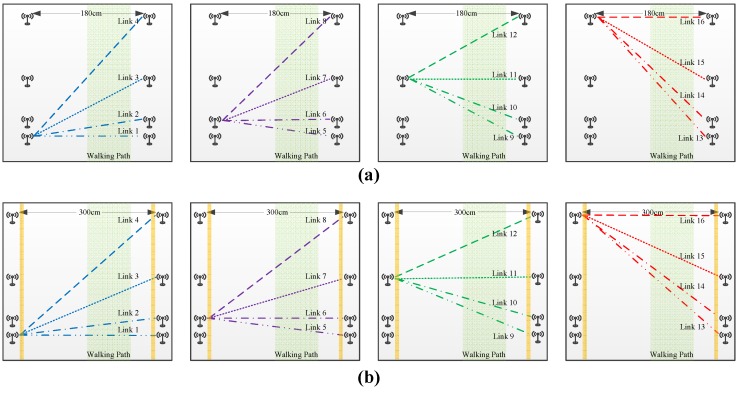
Geography map and link label of each sensing layer. (**a**) LOS scenario; (**b**) NLOS scenario.

According to the above visual analysis of human gait, it can be assumed that walking movements are mainly driven by several parts of body. Therefore, we organized a three-layer vertically deployed RF sensing network (as shown in Figure 1) for the purpose of testing the capability of RF links to sense human biometrics. The same topology was followed in both LOS and NLOS scenarios. The three layers of the sensing network were arranged at 30 cm, 80 cm, and 1.3 m off the ground separately for sensing movements caused by upper limbs, thighs, and lower legs. Each layer of the sensing network consisted of eight RF nodes, as shown in Figure 2. These nodes were symmetrically distributed on both sides of the walking path, and the non-uniform pitches of adjacent nodes were designed for multi-view-angle sampling. The distances between adjacent nodes on each side were 12 cm, 48 cm, and 60 cm. There were a total of 16 effective links in each sensing layer, and each link was parallel to the ground. The implementation of the NLOS scenario was achieved by using several pieces of plank walls with a thickness of 2.5 cm and a height of 2.4 m to obstruct the field of view (FOV) between the flanked RF links. The networks had a bilateral distance of 1.8 m in the LOS scenario and 3 m in the NLOS scenario.

The sensing networks were comprised of the integrated module XM2110 from MEMSIC Inc. (Andover, MA, USA). Each node integrated an ATmega1281 micro-controller (Chandler, AZ, USA) and an AtmelRF230 RF transceiver (Chandler, AZ, USA). With the software configuration based on the TinyOS operating system, each node operated in the 2.4 GHz frequency band. The Zigbee protocol—IEEE 802.15.4 compliant—was used for wireless networking, and is suitable for low data transmission rates and low power consumption. The scanning rate of the sensor network in this article was set to 10 Hz, and a sink node was used to collect the RSS data from all links.

**Figure 3:**
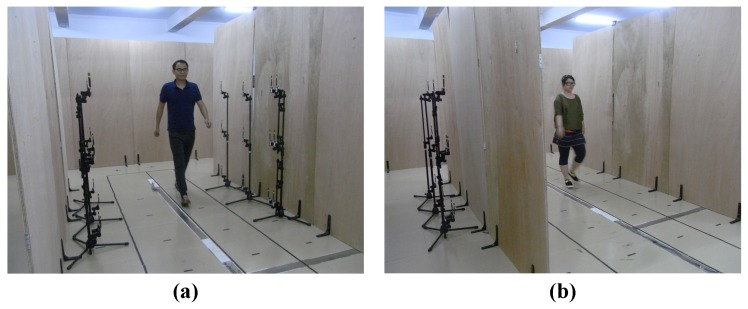
Experimental setup for walker classification in: (**a**) LOS scenario; (**b**) NLOS scenario.

Figure 3 shows the prototype of the proposed sensing method for biometric collection in LOS and NLOS scenarios. Each subject was required to walk on a constrained path with a width of 60 cm. We assumed that the obstructions of the human body will block the direct LOS paths of links and cause varying shadow loss, resulting in fluctuated RSS with R=[R[1],⋯,R[t],⋯,R[T]].

**Figure 4:**
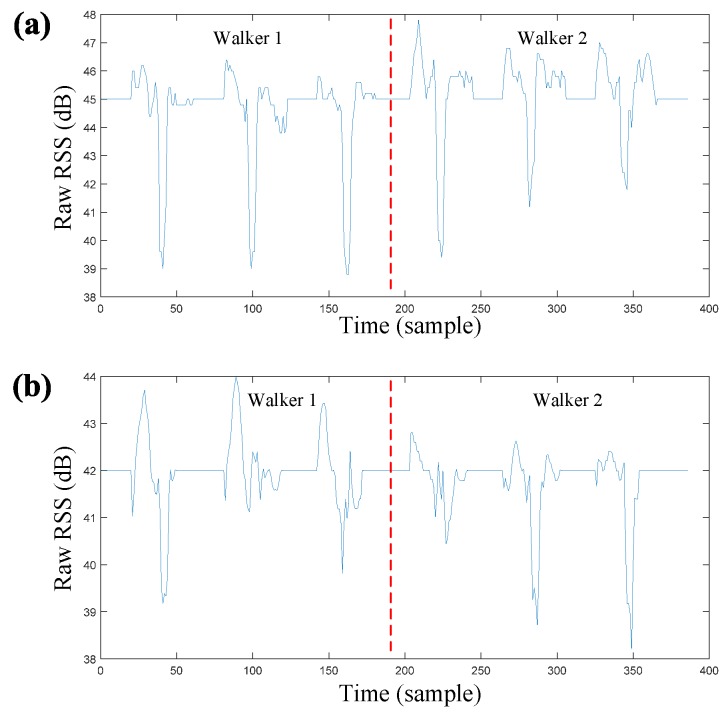
Sensory data streams generated by two individuals. (**a**) LOS scenario; (**b**) NLOS scenario; RSS: received signal strength.

Figure 4 shows the outputs of “Link 1” on the top sensing layer when two subjects traverse the constraint path three times in LOS and NLOS scenarios. The sensory outputs intuitively demonstrate that the vibrations of RSS induced by the two subjects are quite different, while the changing RSSs caused by the same walker seem to be similar in both scenarios. In the following experiments, we will confirm that the feature represented by the temporal RSS values of an arbitrary single RF link can contribute weak discriminative cues for multi-person classification, while the fused feature with multiple links can provide encouraging performance in both LOS and NLOS scenarios.

## 4. VQ- and HMM-Based Walker Classification

**Figure 5:**
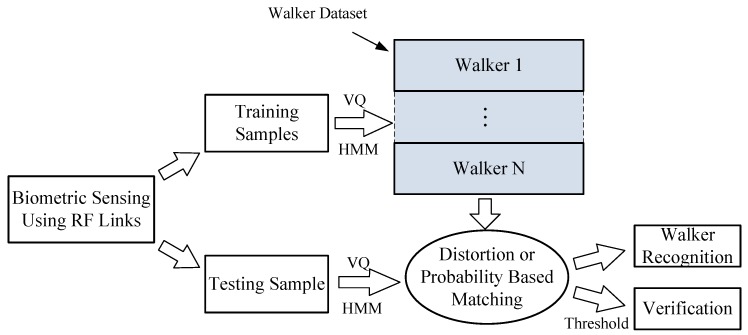
Block diagram of the walker classification process. RF: radio frequency; VQ: vector quantization; HMM: hidden Markov model.

Figure 5 outlines the walker classification process. It is divided into three steps: sensing, training, and testing. In the first step, the temporal RSS data from different combinations of RF links is used for biometric feature representation. In the second step, a codebook and an HMM are built for each registered walker. In the third step, a newly generated sequence is matched against all trained codebooks and HMMs. The associated model with the minimum distortion or maximum likelihood is searched to identify the biometric entity. For the walker verification, the system takes and compares an unknown sequence against the claimed biometric model to obtain a similarity score. By presetting a threshold, the system will make a decision to grant or deny access to an unknown walker. Hence, walker recognition makes a one-to-many matching, while verification involves a one-to-one matching.

### 4.1. VQ-Based Walker Classification

VQ is a widely used tool for lossy data compression [46] and speaker recognition [47]. The effectiveness of VQ for walker classification lies in that the sensory sequences can be encoded by individual codebook. Although the VQ-based classification does not have the ability to model the temporal relationship of sequential signals, it can extract the prototype vectors of a feature from the overall training samples. In the model training process, each codebook is built on each walker’s training data and seeks to minimize the reconstruction distortion. Each codeword in a codebook corresponds to high-frequency data appearing in training samples. Normally, a codebook with moderate size is able to model the identity of a registered walker. If the lightweight VQ-based classification is employed and acceptable accuracy can be achieved, then it will prove the effectiveness of the proposed sensing method.

In particular, we used the sensory RSS gathered from different RF links as the training samples, which can be denoted for short as:

(1) $$R_{w}=\left[R_{w,1},\cdots R_{w,N}\right],$$

where $R_{w}$ refers to the set of collected RSS samples for each walker *w* and *N* is the number of training samples. The *k*-Means algorithm proposed in [48] is employed for the codeword computation due to its simple and efficient implementation. The biometric model of each walker can be represented by a set of codewords, abbreviated as:

(2) $$C_{w}=\left[c_{w,1},\cdots ,c_{w,k},\cdots ,c_{w,K}\right],$$

where $C_{w}$ is the codebook of walker *w*, $c_{w,k}$ is a codeword, and *K* is the size of the codebook.

After the model training process, the recognition of an unknown biometric entity $R_{unknown}$ will be executed by the following distortion assessment:

(3) $$d\left(R_{unknown},C_{w}\right)=\sum_{t=1:T}∥R_{unknown}\left[t\right]-{\hat{c}}_{w,k}∥,$$

where ${\hat{c}}_{w,k}$ is the codeword closest to the vector $R_{unknown}\left[t\right]$ in the codebook $C_{w}$. Then, the identity can be obtained by the following search:

(4) $$\hat{w}=\underset{1\leq w\leq W}{\arg\min}\;d\left(R_{unknown},C_{w}\right).$$

For walker verification, a one-to-one comparison is made:

(5) $$R_{unknown}\in \mathrm{ID}\left\{\begin{array}{cc} Accept & d\left(R_{unknown},C_{ID}\right)<Threshold_{VQ},\\ Reject & Others. \end{array}\right.$$

Only the codebook with claimed identity $C_{ID}$ is used for walker verification. The reconstructed distortion $d(R_{unknown},C_{ID})$ is used as a certification score, and a fixed hard threshold $Threshold_{VQ}$ is set for granting or denying an unknown walker.

### 4.2. HMM-Based Walker Classification

For walker classification, it is obvious that the identity cannot be found directly from the knowledge of sensory RSS sequence. The optimal match can only be searched in a certain probabilistic sense. As a supervised sequential-observation learning method, HMMs have been successfully applied to temporal data modeling such as speech recognition [49] and motion identification [50]. Since the temporal evolution of walking is presented by a sequence of RSSs, we can employ HMM for modeling the temporal transition process of movements so as to robustly recognize an identity.

For the walker classification problem, we establish one HMM with Gaussian mixture emissions for each registered subject, which is denoted compactly by $N_{H}$, $M_{G}$, and χ=(A,B,Π). Here, $N_{H}$ is the number of hidden states and $M_{G}$ is the number of Gaussian models. The transition probabilities matrix of hidden states $A=\{a_{ij}\}$ is defined as:

(6) $$a_{ij}=P\left(q_{t+1}=j|q_{t}=i\right),\;1\leq i,j\leq N_{H},$$

where *i* and *j* are the labels of the hidden states, and $q_{t}$ is the hidden state at time *t* . The transition probability distribution should satisfy the constraints $a_{ij}\geq 0$ and $\Sigma_{j}^{N}a_{ij}=1$, and expresses the degree of relevance between adjacent hidden states. The probability density distribution of the observed vector is defined by $B=\{b_{i}\left(R\left[t\right]\right)\}$ and:

(7) $$b_{i}\left(R\left[t\right]\right)=P\left(R\left[t\right]|q_{t}=i\right)=\Sigma_{m=1}^{M_{G}}c_{im}N\left(\mu_{im},\;\Sigma_{im},\;R\left[t\right]\right),$$

where $c_{im}$ is the mixture weight for the *m*th Gaussian model in state *i*. N is the Gaussian probability density function with mean vector $\mu_{im}$ and covariance matrix $\Sigma_{im}$. The weighting coefficients should satisfy the constraints $c_{im}\geq 0$ and $\Sigma_{m=1}^{M_{G}}c_{im}=1$. The probability density distribution B presents the degree of relevance between the observations and hidden states. The coefficients in the Gaussian models can be estimated using maximum likelihood estimation (MLE) [51]. $\Pi =\{\pi_{i}\}$ is defined as the initial state probability vector with $\pi_{i}=P\left\{q_{1}=i\right\}$, and satisfies the constraints $\pi_{i}\geq 0$ and $\Sigma_{i=1}^{N}\pi_{i}=1$.

For the problem of learning the model parameters χ of an HMM, we first collect several temporal RSS samples as training data and then use the Baum–Welch method to re-estimate the model parameters [52]. For recognizing an unknown sensory sequence $R_{unknown}$, we can calculate the probability $P(R_{unknown}|\chi )$ following the forward–backward algorithm [53].

For an HMM database with *W* registered individuals, the recognition of an unknown walker with $R_{unknown}$ is executed by the following search:

(8) $$\hat{w}=\underset{1\leq w\leq W}{\arg\max}\;P\left(R_{unknown}|\chi_{w}\right).$$

For the walker verification, a similar framework can be followed as:

(9) $$R_{unknown}\in \mathrm{ID}\left\{\begin{array}{cc} Accept & P\left(R_{unknown}|\chi_{ID}\right)>Threshold_{HMM},\\ Reject & Others. \end{array}\right.$$

Here, $Threshold_{HMM}$ is a fixed hard threshold for rejecting or accepting an unknown walker with claimed identity.

## 5. Experiment and Results

In order to demonstrate the proposed sensing method, we conducted a validation experiment in a laboratory environment. A total of 15 subjects participated in our data collection, including five females and ten males. The height of individuals ranged from 155 cm to 186 cm, and the weights were from 48 kg to 80 kg. In the enrollment phase, the sensing network operated on an idle channel, and each participant was required to repeat walking along the specified constrained path 20 times in both LOS and NLOS scenarios, as shown in Figure 3. Every walk was at a self-select speed and strategy, and all participants dressed in summer clothing. Based on the sampling path designed in this article, the sensing network could record at least one walking cycle including two steps for each pedestrian.

In the training phase, we randomly selected 10 samples from each person’s data for biometric modeling. The remaining 10 sequences were used for test analysis. The following statistical results are based on the average values of 100 cross-validations in close-set classification.

### 5.1. Parameter Determination for VQ and HMM

The most important aspect of VQ-based classification is the determination of the size of the codebook. We used the sensory outputs of all 16 effective RF links from each sensing layer as the fused feature and calculated the average accuracy by changing the size of the codebook . The average correct rate or accuracy is defined as:

(10) $$Average\;Accuracy\;\left(AA_{L}\right)=\frac{Number\;of\;truly\;recognized}{Number\;of\;all\;attempts},\;L\in \left\{Top,\;Middle,\;Bottom\right\}.$$

**Figure 6:**
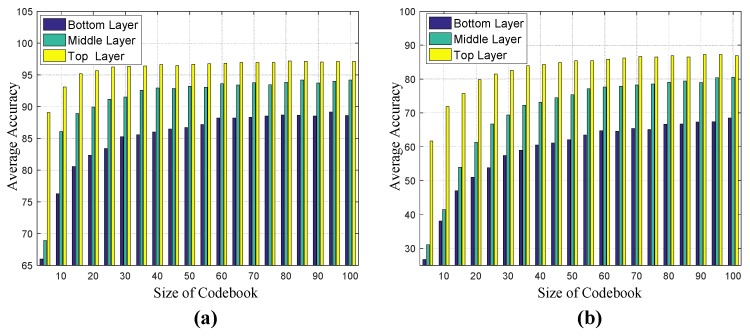
Average correct recognition rates as a function of the size of vector quantization (VQ) codebook for the three-layer sensing networks. (**a**) LOS scenario; (**b**) NLOS scenario.

Figure 6 shows the average results with respect to different codebook sizes. It turns out that when the size of codebook increases to 40, the growth of average accuracy tends to be steady in both LOS and NLOS scenarios. Although the larger codebook sizes will improve accuracy, the computation cost will be much higher. Therefore, for the following analysis, all the VQ-related results are based on the codebook size of 40 unless otherwise stated.

For the HMM-based walker classification, two parameters need to be determined: one is the number of hidden states, and the another is the number of Gaussian models. In this regard, we still use the sensory data generated by all RF links from each layer as the features, and calculate the average recognition rate by changing the two parameters, respectively. The average accuracy of the three-layer sensing networks is used to confirm the optimal parameters of HMMs, which can be denoted as:

(11) $$AA_{average}=\frac{1}{3}\left(AA_{Top}+AA_{Middle}+AA_{Bottom}\right).$$

**Figure 7:**
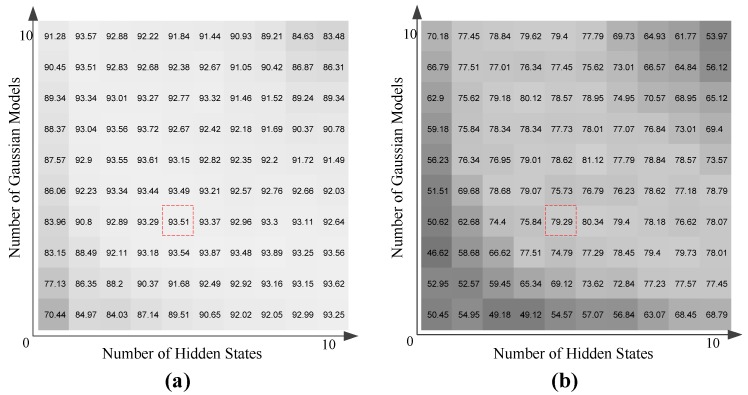
Average correct recognition rates as a function of the number of hidden states and Gaussian models in hidden Markov model (HMM). (**a**) LOS scenario; (**b**) NLOS scenario.

Figure 7 shows the average results with respect to different numbers of hidden states and Gaussian models. Based on the two results in LOS and NLOS scenarios, it is found that when the number of hidden states reaches five and the number of Gaussian models increases to four, the average accuracy grows gradually. Larger numbers of hidden states and Gaussian models may be useful for more accurate modeling, but the computational cost will be much higher and the over-fitting problem may occur.

### 5.2. Recognition Performance with Different Combinations of Links

**Figure 8:**
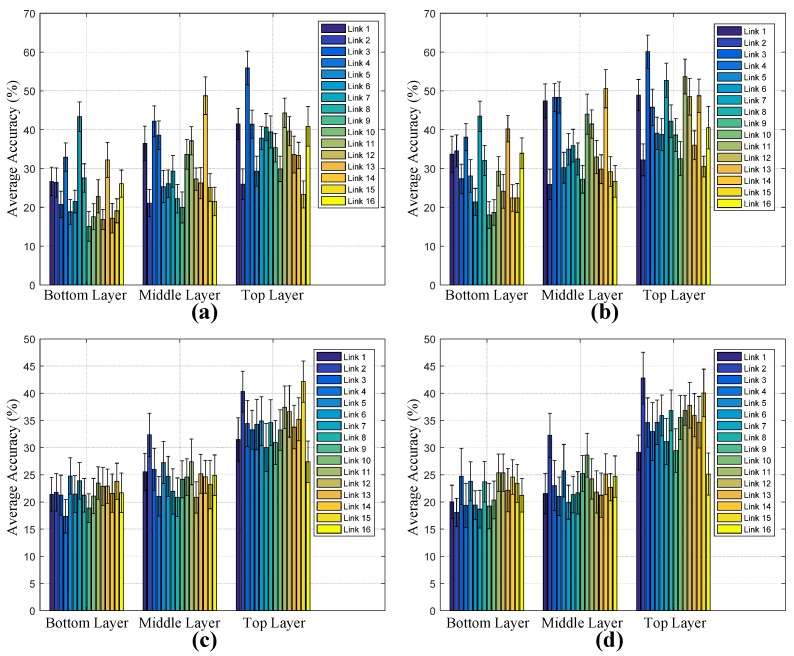
Average accuracy and standard deviation with respect to different single links. (**a**) VQ with LOS; (**b**) HMM with LOS; (**c**) VQ with NLOS; (**d**) HMM with NLOS.

We first use the sensory data of a single RF link from each sensing layer for walker recognition. Figure 8 shows the results involving VQ and HMMs for LOS and NLOS scenarios. It can be found from the results that the average accuracy by using a single link is not satisfactory, regardless of which layer of sensing networks and what feature modeling methods are used. For the recognition in the LOS scenario, the average accuracy is approximately in the range 15–60%, while, in the NLOS scenario, the average accuracy is 15–45%. We can conclude that the biometric sensing capability of a single RF link is limited for multiple-walker recognition.

**Figure 9:**
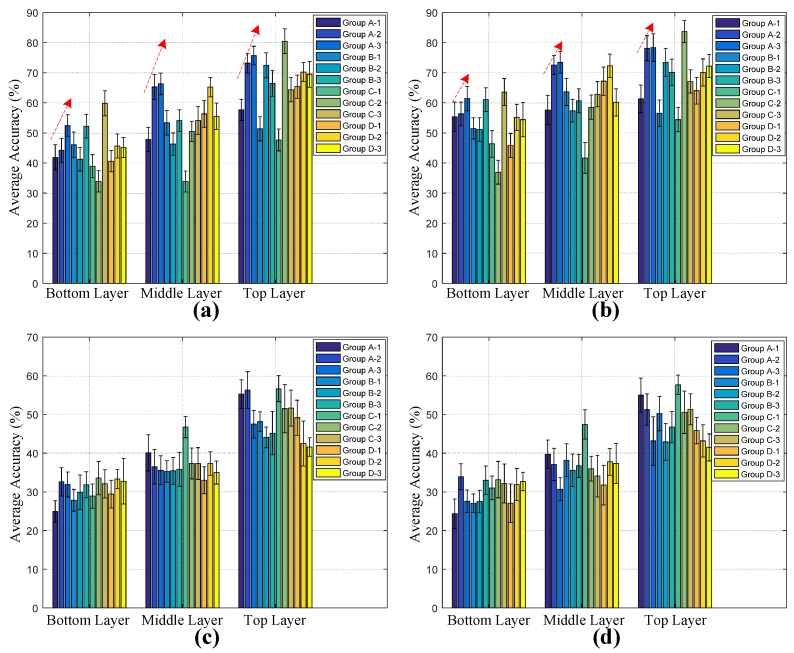
Average accuracy and standard deviation with respect to different dual-links. (**a**) VQ with LOS; (**b**) HMM with LOS; (**c**) VQ with NLOS; (**d**) HMM with NLOS.

We then use the sensory outputs generated by typical dual-link as the biometric features and calculate the average accuracy. Some typical groups of dual-link and geometries are listed in Table 1. There are four groups of the most common geometries to be evaluated. “Group A” indicates that the common node of the two links are close to the constrained path. “Group B” extracts the sensory data with shared node of the two links far away from the walking path being monitored. “Group C” refers to the two links that cross through the constrained path. The angles between the dual-link combinations increase in turn. In “Group D”, the biometric information is obtained by using double links with parallel geometry, in which the distance between the two links increases sequentially. Figure 9 shows the average accuracy and standard deviation when the four groups of dual-links are used for biometric sensing, and three obvious results can be seen. First, compared to the performance of single link sensing in Figure 8, the use of dual-link sensing will significantly improve the accuracy of walker recognition. Second, it is clear that the top layer and middle layer of the sensing networks provide more discriminative biometric features, and the features acquired in the LOS scenario are more reliable than that in the NLOS scenario. Third, in the LOS scenario, the average accuracies of “Group A” increase when the angle of dual-link grows from $3.81^{\circ }$ to $33.69^{\circ }$. Based on the HMM modeling, we can get the highest accuracies with 83.66% and 57.67% in LOS and NLOS environments, respectively.

**Figure 10:**
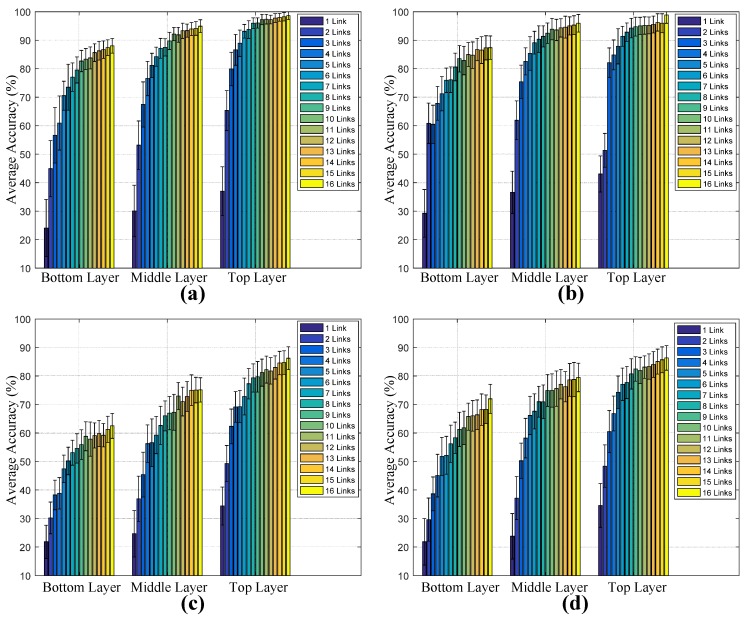
Average correct recognition rates as a function of the number of fused links. (**a**) VQ with LOS; (**b**) HMM with LOS; (**c**) VQ with NLOS; (**d**) HMM with NLOS.

To improve the biometric sensing capability, we increased the number of RF links to sample more informative biometrics. Figure 10 shows the average accuracy and standard deviation based on the features fused by different numbers of RF links in three sensing layers. These different link combinations were randomly selected from the overall 16 links. Three obvious trends can be found: firstly, the recognition accuracy increased with the growth of the number of fused links. Secondly, under the same recognition algorithms and the number of links, the acquired biometric feature from the LOS scenario was more reliable than that from the NLOS scenario. Similarly, the top and middle sensing layers contributed more reliable biometric features than the bottom layer. In addition, from the comparison of overall results, we can see that HMM-based recognition outperformed the VQ approach when fewer RF links were fused. However, the VQ and HMM recognition methods had nearly the same performance when the number of involved links grew from 5 to 16. The reason might be that VQ-based modeling only seeks to minimize reconstruction distortion for training samples, and each registered subject is represented by a scalar codebook. Meanwhile, the HMMs contain informative statistics of temporal relationships from training sequences, which contributes to making a more reliable recognition with respect to fewer available RF links. Throughout all biometric sensing schemes, the maximum accuracies based on VQ are 97.62% and 86.25% in LOS and NLOS scenarios, and 98.63% and 86.33% when HMMs are employed.

### 5.3. Walker Verification

For walker verification, we fused the sensory outputs of all 16 links from three sensing layers as the biometric feature. During the testing phase, a walker to be identified performed a one-to-one model matching with the claimed identity. Two commonly used types of errors were employed for performance analysis, which can be defined as follows:

(12) $$\begin{array}{cc} False-Acceptance\;Rate\;(FAR) & =\frac{Number\;of\;accepting\;an\;impostor\;walker}{Number\;of\;impostor\;attempts},\\ False-Rejection\;Rate\;(FRR) & =\frac{Number\;of\;rejecting\;a\;legitimate\;walker}{Number\;of\;legitimate\;attempts}. \end{array}$$

For the VQ- and HMM-based modeling methods, we can regard the outputs of the reconstructed distortion and the maximum likelihood estimation, respectively, as the scores of similarity. By introducing the changing hard thresholds for decision making, the walker verification will be cast into two-class recognition problems.

**Figure 11:**
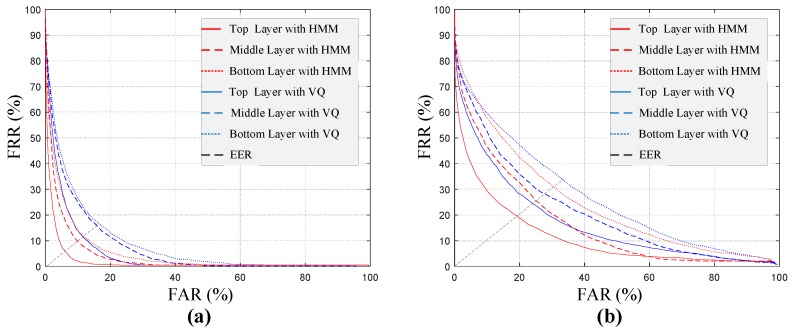
Detection error tradeoff (DET) curves of walker verification in: (**a**) LOS scenario; (**b**) NLOS scenario. EER: equal error rate.

Figure 11 shows the detection error tradeoff (DET) curves with respect to the fused features sampled from three sensing layers in LOS and NLOS environments. The curves closer to the origin usually represent the better performance when the associated sensing feature and modeling method are used. The equal error rate (EER)—which is defined as the value when FAR and FRR are equal—is introduced for performance comparison. Table 2 shows the EER results. Throughout the results in LOS and NLOS environments, the HMM-based walker verification was more robust than the VQ method, and the top sensing layer provided the most reliable biometric information.

The relevant algorithms in our experimental studies ran on an Intel Core i3-6100 3.7 GHz computer (Santa Clara, CA, USA) by Matlab codes (Version: R2016a, MathWorks, Natick, MA, USA). The average time spent on a walker verification was 3.6 ms with maximum 12.8 ms with VQ, while the average time using HMM was 27.30 ms with a maximum 126.29 ms.

## 6. Conclusions

This article explores the novel use of multiple narrowband RF links to sample biometric traits generated by walking on a constrained path. We proposed a three-layer RF sensing networks for sampling biometric features generated by upper limbs, thighs, and lower legs. Different sensing layers, different numbers of fused RF links, and different modeling algorithms were studied to improve classification accuracy. Better performance could be obtained by using multiple RF links located at heights of upper limb and deploying the sensing network in LOS.

The proposed sensing method has three main advantages. First, the sensory data sampled by multiple links provide more robust and stable human motion cues, which can capture richer discriminative signatures and contribute to performance improvement for walker classification tasks. Second, the proposed sensing method can explore the existing wireless communication measurements, hardware, and facilities to achieve the ubiquitous biometric sensing. Definitively, this work can boost a coverage-scalable, easy-constructed and energy-saving RF sensing networks for biometric applications. Third, under the motion-specific RF sensing paradigm, the features can be directly encoded into low-dimensional measurement, and thus the operations for recognition tasks can be performed directly on measurement space, which can facilitate the development of real-time multi-person tracking and security systems.

Although the experimental results have demonstrated the effectiveness of our proposed approach, the systematic design for acquiring biometrics generated by walking is still at its initial development stage. There are a lot of challenging problems that need to be addressed. One potential improvement is how to explore new sensing methods towards energy-saving system. The compressive sensing (CS) based data acquisition and the passive network activation by sensory fusion of a variety of signal modalities (e.g., pyroelectric infrared (PIR) sensors) are possible ways for building energy-efficient system. Other research questions are whether and how the length of the walk will influence the results. Based on the sampling path designed in this article, the sensing network could record at least one walking cycle including two steps for each pedestrian. We will plan to analyze this influence in future research. In addition, the new classification algorithms and multimodal biometric fusions are expected for the open-set, cloth-independent, and simultaneous multiple human recognition.

## Figures and Tables

**Table 1:** Typical groups of dual-link and geometries.

		Group A			Group B			Group C			Group D		
	No.	Geometry	Links	Angle	Geometry	Links	Angle	Geometry	Links	Angle	Geometry	Links	Distance
			(label)	(degree)		(label)	(degree)		(label)	(degree)		(label)	(cm)
LOS	1	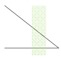	{1,2}	$3.81^{{}^{\circ}}$	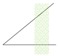	{1,5}	$3.81^{{}^{\circ}}$	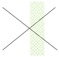	{2,5}	$7.63^{{}^{\circ}}$	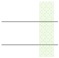	{1,6}	12
	2		{1,3}	$18.43^{{}^{\circ}}$		{1,9}	$18.43^{{}^{\circ}}$		{3,9}	$36.87^{{}^{\circ}}$		{1,11}	60
	3		{1,4}	$33.69^{{}^{\circ}}$		{1,13}	$33.69^{{}^{\circ}}$		{4,13}	$67.38^{{}^{\circ}}$		{1,16}	120
NLOS	1		{1,2}	$2.29^{{}^{\circ}}$		{1,5}	$2.29^{{}^{\circ}}$		{2,5}	$4.58^{{}^{\circ}}$		{1,6}	12
	2		{1,3}	$11.31^{{}^{\circ}}$		{1,9}	$11.31^{{}^{\circ}}$		{3,9}	$22.62^{{}^{\circ}}$		{1,11}	60
	3		{1,4}	$21.80^{{}^{\circ}}$		{1,13}	$21.80^{{}^{\circ}}$		{4,13}	$43.60^{{}^{\circ}}$		{1,16}	120

LOS: line-of-sight; NLOS: non-line-of-sight.

**Table 2:** Equal error rates (EERs) with respect to different sensing layers and different verification algorithms.

	**LOS**		**NLOS**	
	VQ	HMM	VQ	HMM
Top	11.72%	6.14%	24.32%	19.36%
Middle	15.88%	9.84%	28.01%	25.31%
Bottom	16.62%	11.75%	33.51%	30.74%

VQ: vector quantization; HMM: hidden Markov model.
